# Classification of peanut pod rot based on improved YOLOv5s

**DOI:** 10.3389/fpls.2024.1364185

**Published:** 2024-04-15

**Authors:** Yu Liu, Xiukun Li, Yiming Fan, Lifeng Liu, Limin Shao, Geng Yan, Yuhong Geng, Yi Zhang

**Affiliations:** ^1^ Hebei Agricultural University, Baoding, China; ^2^ State Key Laboratory of North China Crop Improvement and Regulation, Baoding, China; ^3^ Technology Innovation Center of Intelligent Agricultural Equipment, Baoding, China

**Keywords:** peanut pod rot, machine vision, improved YOLOv5s, Shuffle Attention, grading classification

## Abstract

Peanut pod rot is one of the major plant diseases affecting peanut production and quality over China, which causes large productivity losses and is challenging to control. To improve the disease resistance of peanuts, breeding is one significant strategy. Crucial preventative and management measures include grading peanut pod rot and screening high-contributed genes that are highly resistant to pod rot should be carried out. A machine vision-based grading approach for individual cases of peanut pod rot was proposed in this study, which avoids time-consuming, labor-intensive, and inaccurate manual categorization and provides dependable technical assistance for breeding studies and peanut pod rot resistance. The Shuffle Attention module has been added to the YOLOv5s (You Only Look Once version 5 small) feature extraction backbone network to overcome occlusion, overlap, and adhesions in complex backgrounds. Additionally, to reduce missing and false identification of peanut pods, the loss function CIoU (Complete Intersection over Union) was replaced with EIoU (Enhanced Intersection over Union). The recognition results can be further improved by introducing grade classification module, which can read the information from the identified RGB images and output data like numbers of non-rotted and rotten peanut pods, the rotten pod rate, and the pod rot grade. The Precision value of the improved YOLOv5s reached 93.8%, which was 7.8%, 8.4%, and 7.3% higher than YOLOv5s, YOLOv8n, and YOLOv8s, respectively; the mAP (mean Average Precision) value was 92.4%, which increased by 6.7%, 7.7%, and 6.5%, respectively. Improved YOLOv5s has an average improvement of 6.26% over YOLOv5s in terms of recognition accuracy: that was 95.7% for non-rotted peanut pods and 90.8% for rotten peanut pods. This article presented a machine vision- based grade classification method for peanut pod rot, which offered technological guidance for selecting high-quality cultivars with high resistance to pod rot in peanut.

## 1 Introduction

Peanut pod rot, also known as fruit rot, significantly impacts peanut yield and quality, with occurrences noted in the United States (Wheeler et al., 2016), Egypt (Elsayed Abdalla and Abdel-Fattah, 2000) and various regions of China, including Shandong (Zhang et al., 2016) and Hebei Province (Li et al., 2011). The disease’s prevalence and severity are leading to increased losses annually, with affected plots seeing up to a 15% yield reduction and severely infected areas losing up to 50%. In some cases, it can lead to total crop failure (He et al., 2022). So far, *N. vasinfect* (Gai et al., 2011; Sun et al., 2011), *Fusarium* sp (Liu et al., 2020), *N. striata* (Sun et al., 2012), *P. myriotylum* (Yu et al., 2019), and *R. solani* (Chi et al., 2015) have been identified as the pathogenic bacteria of peanut pod rot in China. Peanut pod rot poses a severe danger to the safety of peanut output and quality, and it is critical to strengthen effective prevention and control of it.

The difficulty in preventing and treating peanut pod rot can be attributed to the wide range of pathogen hosts (Abd El-aal et al., 2013) and the current lack of varietal resistance (Walker and Csinos, 1980; Lewis and Filonow, 1990; Besler et al., 2003). Varietal resistance is frequently improved through breeding, which is an efficient method of preventing peanut pod rot (Wynne et al., 1991). By assessing the resistance grade of individual peanut plants to pod rot, superior germplasm can be identified, facilitating the development of new peanut varieties. There is comparatively little research on peanut pod rot in China, with the majority of studies on the pathology of peanuts being on leaf diseases, bacterial wilt, and web blotch. At present, the grade classification of individual peanut pod rot is still usually done manually. Manual categorization is labor-intensive, time-consuming, and prone to errors like misidentification, abandonment, and repeated recognition as work time grows, which is thus not ideal for large-scale grading because of the varied grades of peanut decay. More precise grade classification can be attained by machine vision, which can precisely identify and interpret illness signs in photos, extract important information from them, classify and assess them in accordance with predetermined criteria. Additionally, machine vision technology can expedite breeding operations by increasing the speed and efficiency of grade classification in comparison to manual categorization.

CNN (Convolutional neural network) has recently achieved substantial results in the field of object identification (Zaidi et al., 2022), including Faster R-CNN (Ren et al., 2017), YOLO (Redmon et al., 2016), SSD (Single Shot MultiBox Detector) (Liu et al., 2016), etc. Crop identification based on machine vision is more efficient and less expensive, exhibiting a progressive trend of replacing manual identification. Machine vision models have excelled in crop disease detection. Habib et al. (2020) achieved over 90% accuracy in classifying papaya diseases using K-means clustering for segmentation and support vector machines for identification. Harakannanavar et al. (2022) improved this technique by extracting tomato leaf boundaries with K-means clustering and contour tracing, employing SVM (Support Vector Machine), CNN, and K-NN (K-Nearest Neighbors) algorithms for classification, with CNN attaining an impressive 99.6% accuracy rate. Hua et al. (2022) introduced a PD R-CNN algorithm for crop disease detection that incorporates multi-feature decision fusion, consistently delivering accuracy rates above 85% across various disease types. In citrus orchards, Pydipati et al. (2005) developed an algorithm using the CCM (Color Co-occurrence Method) combined with Mahalanobis distance-based and neural network classifiers, achieving over 95% accuracy in distinguishing between healthy and diseased citrus leaves by leveraging hue and saturation features. To address the challenge of diagnosing visually similar corn diseases in the field, He et al. (2023) enhanced the Faster R-CNN by integrating batch normalization and a central loss function, resulting in a model that surpassed the original Faster R-CNN and SSD in terms of average recall rate, F1 score, and both accuracy and detection speed. While these algorithms excel at identifying and labeling lesions, they do not quantify the number of lesions or provide crop counts. Our study addresses this gap by utilizing the YOLO series algorithm, renowned for its object detection capabilities, to recognize peanut images.

The use of YOLO algorithms in agriculture is now a comparatively developed technique. By introducing light-weighting enhancements to YOLOv3, Shen and Zhao (2021) developed a peanut seed identification model with great accuracy that can operate in real-time on the CPU. By adding DenseNet interlayer density, Gai et al. (2023) enhanced the feature extraction ability of the YOLOv4 backbone network CSPDarknet53. Sozzi et al. (2022) tested six versions of the original YOLO model, and the results demonstrated that YOLOv5s can identify green grapes quickly and accurately. Lawal (2023) upgraded the YOLOv5 backbone and neck networks and changed the loss function to EIoU to improve the robustness in complicated and ever-changing situations. Lawal (2021) improved the YOLOv3 model to solve interference problems such as branch and leaf obstruction, lighting shifts, and fruit overlapping. In the identification application of tomatoes, the improved YOLOv3 model exhibited an average prediction rate of 99.5%. Aran et al. (2016) employed a BPNN (Back-propagation neural network) for the grade classification of cashews, reaching an accuracy of 96.8%.

These methodologies can be well coupled with machine vision in their respective crop fields, providing technological backing for the feasibility of this study. The primary challenge faced in this study was to reduce the model size while maintaining recognition performance, in order to adapt it for embedded systems and enable effective grading of outdoor peanut pod rot. The challenges include the scarcity and diversity of data, which complicate the collection of standardized datasets and model training; the complexity of peanut pod rot features, especially the high variability at different stages, presents significant difficulties for accurate identification and grading; although existing machine vision models perform excellently in several other domains, specific improvements are still required to enhance performance for the characteristics of peanut pod rot.

There is currently no research on grading peanut pod rot using machine vision. This study aims to integrate lightweight object detection models into portable devices to support field applications. Given the high computational resource demands, YOLOv8 is not suitable for mobile or embedded devices with limited computing power. In contrast, the YOLOv5 series of algorithms, with their smaller size, are more suitable for integration into such embedded systems. Among the various versions of YOLOv5, the YOLOv5s has the smallest model size, with a 35% and 70% reduction in size compared to YOLOv8s and YOLOv8n, respectively, making YOLOv5s an ideal choice for integration into resource-constrained devices. To enhance the data reliability and work efficiency, the future approach to image acquisition will shift from single-plant per image to multiple-plants per image, guiding the detection task towards small object detection. With its multi-scale feature fusion, optimized anchoring mechanism, powerful data augmentation, and highly customizable architecture, YOLOv5s has proven to improve the precision of small object detection while maintaining rapid processing speed. Based on these factors, the model was selected for optimization to meet the needs of practical applications.

To facilitate the screening of peanut germplasm resources resistant to pod rot, this paper proposed a grading algorithm based on Shuffle Attention and prediction box location optimization, targeting interference such as peanut pod adhesion, root stem and leaf occlusion. To begin, using the YOLOv5s identification model, the Shuffle Attention mechanism was used to improve the capability of feature representation, location accuracy of lesion area, and robustness in complex backdrops. Then, the loss function was enhanced to improve the regression accuracy of the prediction box and reduce the likelihood of errors and omissions. Finally, the rotten pod rate was estimated by calculating the quantity of rotten peanut pods according to the projected results. The grade classification was carried out based on the rotten pod rate and the results were further compared with those of YOLOv5s, YOLOv8n, and YOLOv8s models. Based on this, the efficiency of the proposed method in this study can be verified.

The rest of this work is structured as follows: Section 2 discusses the planting environment of peanuts, the establishment procedure of the dataset, and the design and optimization of the pod rot grading model. Section 3 introduces relevant tests and compares the recognition and prediction performance of four models. Section 4 discusses the shortcomings of the proposed method and future research directions for the grade classification of peanut pod rot. Section 5 highlights the experimental results of the proposed model, emphasizing the application value of this study.

## 2 Materials and methods

### 2.1 Sample acquisition

The samples were collected from the Experimental Station of Hebei Agricultural University in Qingyuan District, Baoding City, Hebei Province (38°80’N, 115°57’E). A cultivar of peanut, Jinongxian No.1, was taken as the experimental sample in this study, which was planted in spring, 2023, with ridge plastic film and mulching, ridge spacing of 85 cm and two rows per ridge. The average row spacing was 42.5 cm, with a hole spacing of 15.5 cm and two seeds per hole. The planting density was 60750 holes/acre.

Thirty peanuts were taken as samples from the field to the laboratory for washing to remove soil on surfaces. To acquire the dataset, pictures were taken using a SAMSUNG Galaxy S20+ phone with 64 megapixels. The sampling period was set from September 27th to September 29th, 2023, all of which are sunny days. The shooting time was set from 12:00 to 14:00 with sufficient light and 16:00 to 18:00 with dim light. All pictures were taken under natural light, and a total of 2000 peanut images were collected. The shooting angle was set as either top right or side up, while the shooting distance was set as long shot, close shot, and ultra-close shot. The distance from peanuts in the long shot was about 120 cm, the close shot about 40 cm, and the ultra-close shot about 10 cm.

**Figure 1:**
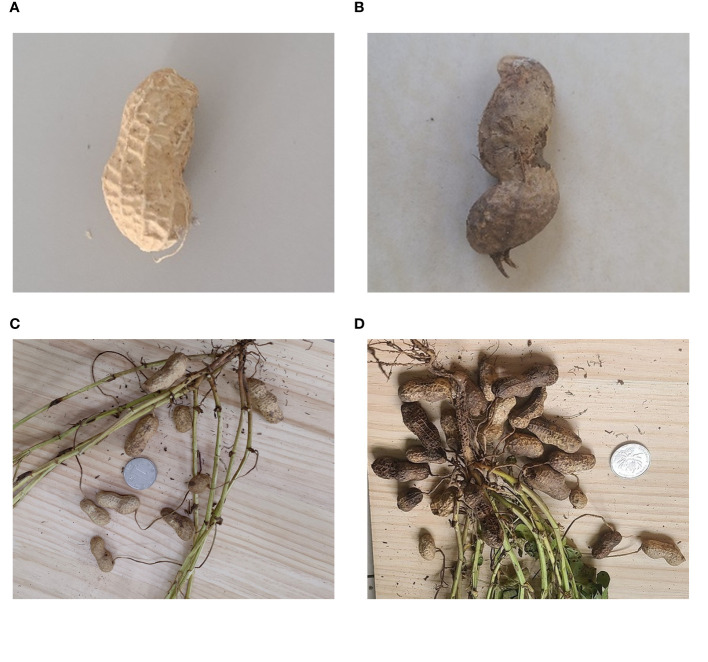
Original peanut image samples. **(A)** Individual non-rotted peanut; **(B)** Individual rotten peanut; **(C)** Low-yielding plant without occlusion and adhesion; **(D)** High-yielding plant with severe occlusion and adhesion.

High-yielding peanut plants tend to stack more frequently because of the abundance of pods, which makes automatic identification challenging. It is unavoidable to run into problems like peanut occlusion and adhesion when taking pictures. Individual peanut and pod images were captured independently to better avoid interference in image recognition and enhance the accuracy and robustness of the model. **Figure 1** presents the images of typical samples.

### 2.2 Dataset production and image enhancement

The images obtained by the phone have a pixel size of 4032*1816. Although a large pixel size can improve the training effect, it significantly affects the training speed. As a result, the pixel size of the original image was resized to be 1400*631.

**Figure 2:**
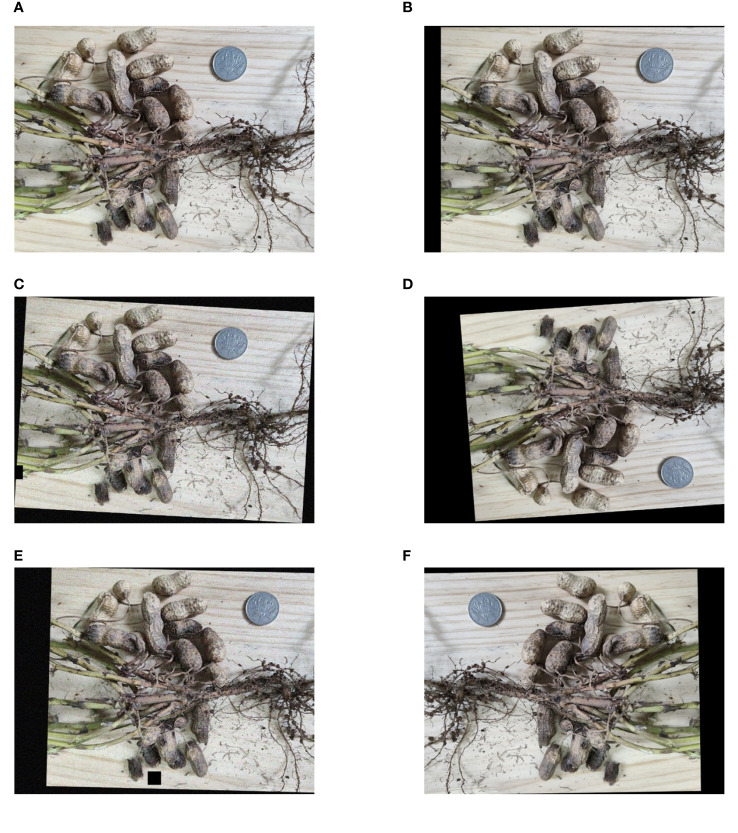
Original and enhanced images. **(A)** Original image; **(B)** Translation; **(C)** Rotation+cutout+noise; **(D)** Vertical flip+rotation+translation; **(E)** Rotation+cutout+noise+translation; **(F)** Horizontal flip+rotation+translation.

Labeling was used to annotate the gathered peanut images. Mark the non-rotted peanuts (G) and rotten peanuts (R) individually throughout labeling, and save the files on the computer in the “xml” format. Before training the object detection model, five enhancement procedures were randomly combined and applied to each image to increase the sample size and boost the training effect. The enhancement treatment included noise addition, cutout, rotation, cropping, translation, horizontal flip, and vertical flip. **Figure 2** depicts the enhanced image. The dataset was finally expanded to 12,000 sheets, which promoted the learning effect of the model on the characteristics of non-rotted and rotten peanuts. There was a total of 83,850 labels in the dataset, including 56,730 non-rotted peanuts and 27,120 rotten peanuts. The dataset was randomly divided into training and testing sets in a 9:1 ratio.

### 2.3 YOLOv5 model

**Figure 3:**
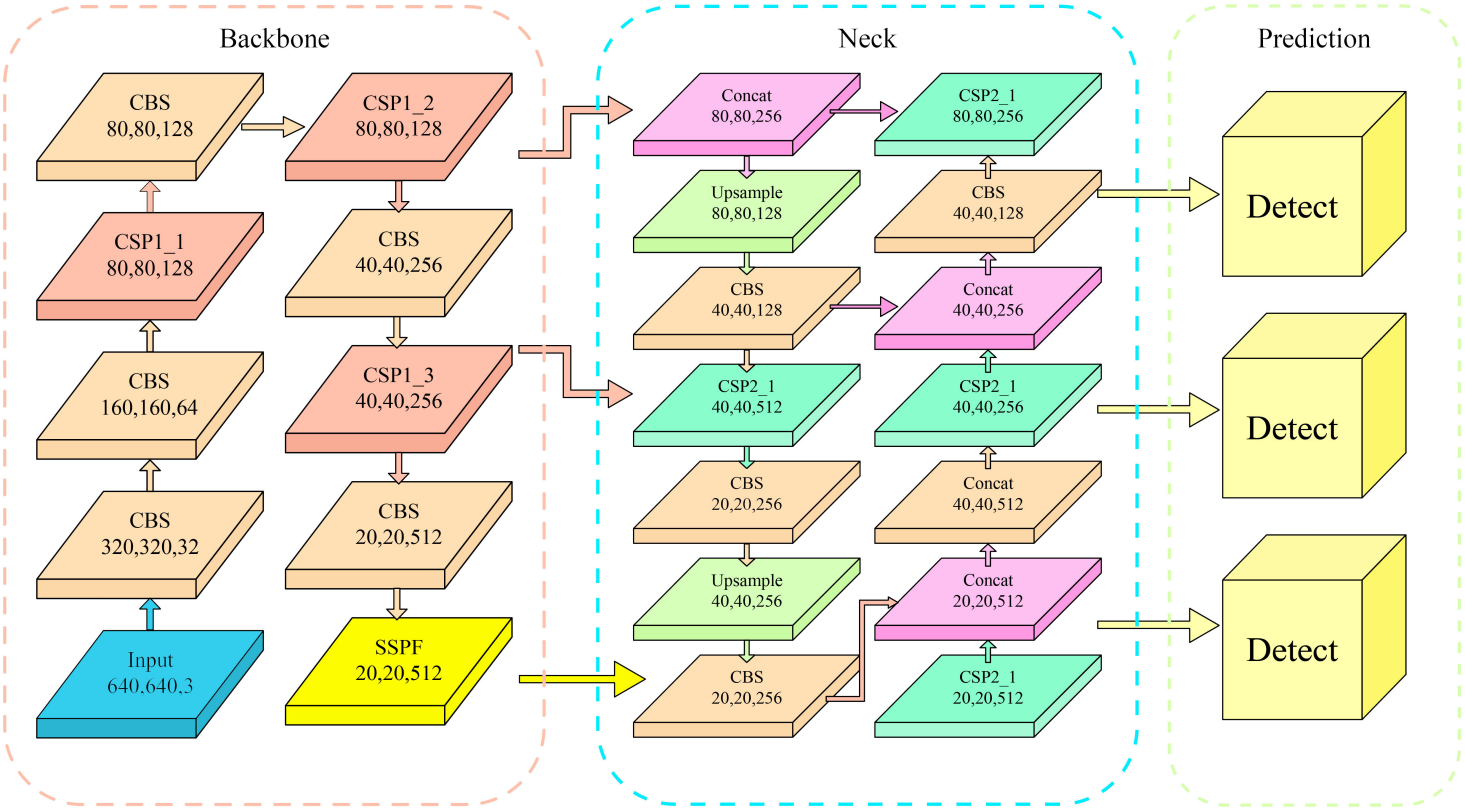
Network architecture diagram of YOLOv5.

The YOLOv5 network structure (Qiao et al., 2021) consists of three main components: Backbone, Neck, and Prediction Head, as shown in **Figure 3**. The Backbone network adopts the CSPDarknet53 architecture, which performs well in feature extraction and was used to extract rich multi-scale features from input images. The feature fusion module was used to fuse feature maps with different scales from the Backbone network. YOLOv5 employed a Feature Pyramid Network (FPN) to fuse features at different levels through upsampling and downsampling, thereby improving the accuracy and robustness of object detection. The Prediction Head was responsible for generating the bounding box and category prediction of the object. YOLOv5 adopted a decoupled multi-level prediction head structure that can effectively handle objects of different scales, achieving a good balance between the speed and accuracy of identification. The combination of these components gave YOLOv5 excellent performance and efficiency in object detection tasks.

### 2.4 Improvement of feature extraction backbone network

This study enhances YOLOv5s to classify the grade of each peanut and calculate the rotten pod rate. It is required to output the total number of G and R labels.

**Figure 4:**
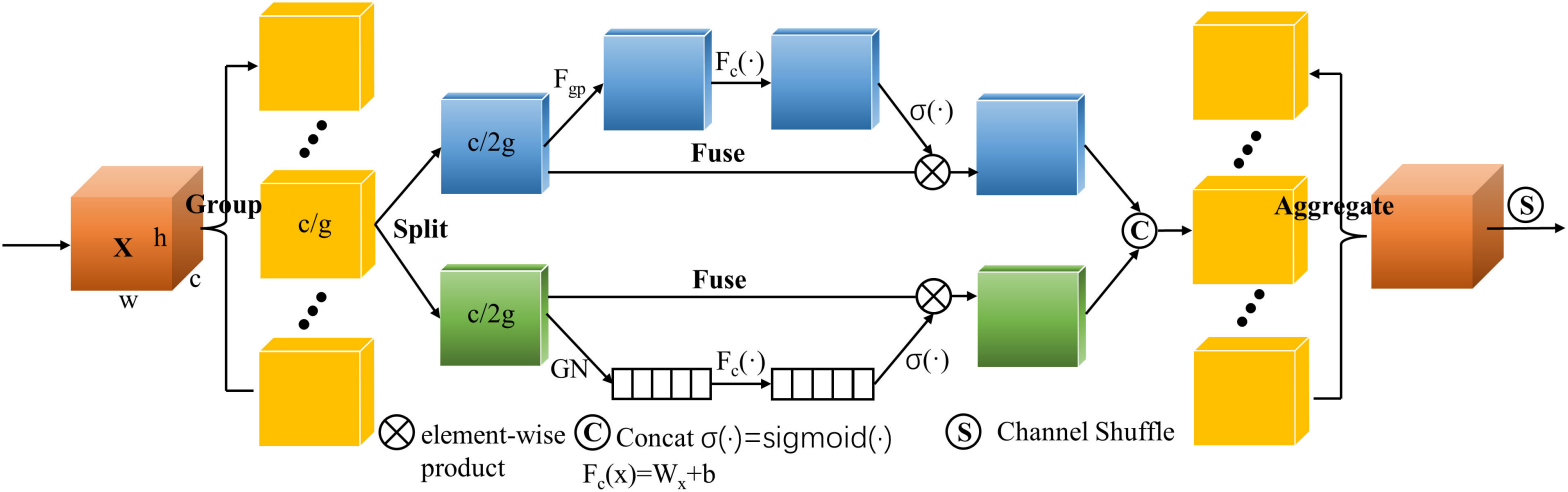
Schematic diagram of Shuffle Attention module.

Some peanuts grow densely and have problems like adhesion and occlusion, which makes it challenging to effectively identify some peanuts separately. Therefore, a Shuffle Attention (SA) module (Zhang and Yang, 2021) was devised in this study. Shuffle Attention is a method of describing feature dependencies through grouping, parallel processing, and information exchange. According to the schematic diagram shown in **Figure 4**, SA first divided the channel dimensions into several subfeatures and processed each subfeature in both spatial and channel dimensions using the Shuffle Unit. The channel shuffle operator was then employed to enhance information exchange between distinct subfeatures after all subfeatures had been summarized. After that, Shuffle Attention was placed after each C3 module in the Backbone, which made local features visible to the attention module. The Shuffle Attention was performed on each layer to share learning pressure.

The purpose of adding the SA module is as follows:

1. Boost the capacity for feature representation. Through channel shuffling and self-attention mechanisms, the SA module can improve the network’s ability to represent features, including long-distance dependency and contextual information. It can also help extract features related to peanut pod rot from images more effectively, such as fine details of lesion areas and contextual information.
2. Improve the positioning accuracy of lesion areas. The SA module employs a self-attention mechanism to gather association information from various positions of the image. Based on this, the lesion area of peanut pod rot can be located more precisely, thereby improving positioning accuracy and minimizing missing and false identification.
3. Enhance the ability to distinguish between non-rotted and rotten peanuts. Peanuts differ from one another in their physical characteristics. The channel shuffling and self-attention mechanism of the SA module can distinguish between rotten and non-rotted peanuts based on minute feature differences. YOLOv5s can learn and discriminate between rotten and non-rotted peanuts, boosting the network’s ability to differentiate pod quality.

**Figure 5:**
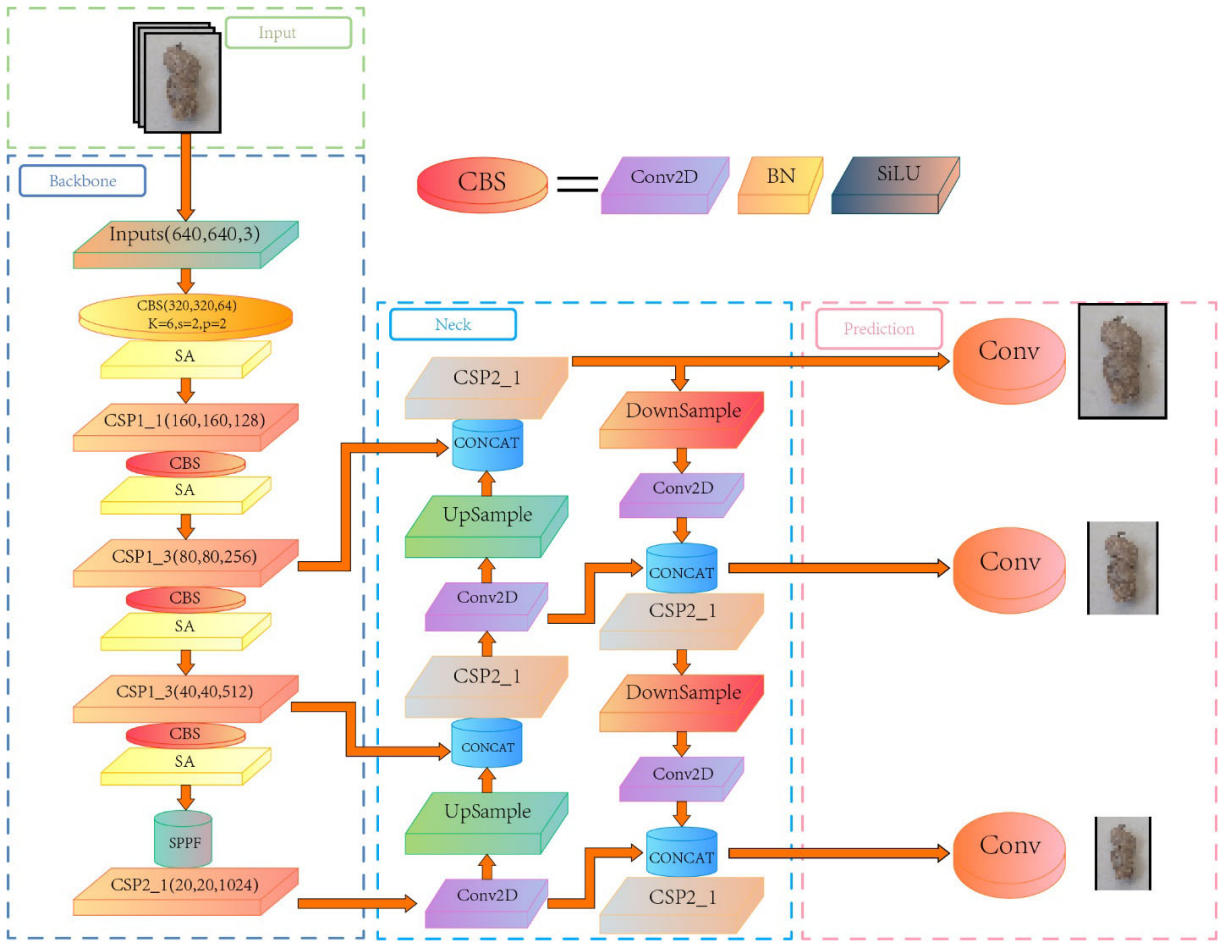
YOLOv5s architecture diagram with added Shuffle Attention module.

It can be concluded that the SA module has increased the feature representation ability, the positioning accuracy of the lesion area, and the capacity to discriminate different disease grades. The introduction of the SA module to YOLOv5s has promoted the accuracy and robustness of peanut pod rot identification by improving the effectiveness of grade classification. **Figure 5** depicts the overall architecture design of adding a SA module to YOLOv5s.

### 2.5 Loss function

The loss functions of YOLOv5s include Classification Loss ( $L_{\text{cla}}$ ), Localization Loss ( $L_{\text{loc}}$ ), and Confidence Loss ( $L_{\text{conf}}$ ). The total loss function is the sum of the three, as shown in Equation (1):


(1) $$Loss=L_{\text{cla}}+L_{\text{loc}}+L_{\text{conf}}$$

Currently, the Localization Loss used in the YOLOv5s model is CIoU (Lu et al., 2022). The sample size of non-rotted peanuts in the dataset was much larger than that of rotten peanuts. The significant quantity difference resulted in a problem of imbalanced samples. Therefore, there is a higher requirement for the accuracy of prediction box regression. The calculation formula for CIoU is as shown in Equations (2)–(4):


(2) $$CIoU=1-IoU+\frac{\rho^{2}\left(b,b^{gt}\right)}{c^{2}}+av$$

(3) $$v=\frac{4}{\pi^{2}}{\left(arctan\frac{w^{gt}}{h^{gt}}-arctan\frac{w}{h}\right)}^{2}$$

(4) $$\alpha =\left\{ \begin{array}{l} 0,\;if\;IoU<0.5\\ \frac{v}{1-IoU+v},\;if\;IoU\geq 0.5 \end{array}\right.$$

Where, IoU refers to the intersection over union between the ground truth box and the prediction box. $\rho^{2}(b,b^{gt})$ refers to the Euclidean distance between the center points of two boxes. $c^{2}$ is the squared≥value of the diagonal length of the minimum closure region that can contain two boxes at the same time. The ratio of the two represents the distance between the ground truth box and the prediction box. av is the influencing factor of the length-width ratio between the two boxes. *w*, *h*, $w^{gt}$ , and $h^{gt}$ represent the width and height of the prediction box and the ground truth box, respectively.

When there is an inclusion phenomenon between the detection box and the ground truth box, CIoU overcomes the problems of degradation to IoU as well as the slow convergence in the horizontal and vertical dimensions when the two boxes cross. Although CIoU offers certain advantages over IoU, the difference in aspect ratio given by v in the formula is not the real difference between width and height and its confidence, which will impede effective similarity optimization of the model.

EIoU takes into account the real difference in length, width, overlapping area, and center point distance (Zhang et al., 2022). It solves the imprecise definition of aspect ratio based on CIoU by calculating the difference in width and height instead of aspect ratio, thus boosting regression accuracy. The imbalance between non-rotted and rotten peanut samples in BBox regression can be resolved by introducing Focal Loss. Therefore, EIoU was used in place of CIoU in this study, and the calculation formula for EIoU is as shown in Equation (5):


(5) $$EIoU=1-IoU+\frac{\rho^{2}(b,b^{gt})}{c^{2}}+\frac{\rho^{2}(w,w^{gt})}{c_{w}^{2}}+\frac{\rho^{2}(h,h^{gt})}{c_{h}^{2}}$$

Where, $c_{w}$ and $c_{h}$ are the width and height of the bounding rectangle of the two boxes, respectively. $\frac{\rho^{2}(w,w^{gt})}{c_{w}^{2}}$ and $\frac{\rho^{2}(h,h^{gt})}{c_{h}^{2}}$ reveal the difference in width and height between the prediction box and the ground truth box.

The improved model is named YOLOv5s-ES, which was established based on the YOLOv5s model with an introduction of the SA module and a replacement of CIoU with EIoU.

### 2.6 Grade classification module

On the one hand, the grade classification of peanut pod rot can be used to determine the severity of diseases. Different stages of the disease may necessitate different prevention and control measures, and the grading aids in the selection of appropriate tactics as well as the improvement of preventative and control effectiveness. On the other hand, the grade classification can offer timely awareness of the disease progression. Taking early response measures is advantageous for sensible resource allocation and cost reductions. The grade classification of peanut pod rot can be claimed to increase targeted and effective prevention and control work, ensure peanut output and quality, and reduce economic losses.

According to the findings of Wheeler et al. (2016), the following are the grading criteria for peanut pod rot: Level 1 for no rotten fruit, with a rotten pod rate of 0; Level 3 for a rotten pod rate between 0 and 10%; Level 5 for a rotten pod rate between 10% and 25%; Level 7 for a rotten pod rate between 25% and 50%; and Level 9 for a rotten pod rate larger than 50%.

**Figure 6:**
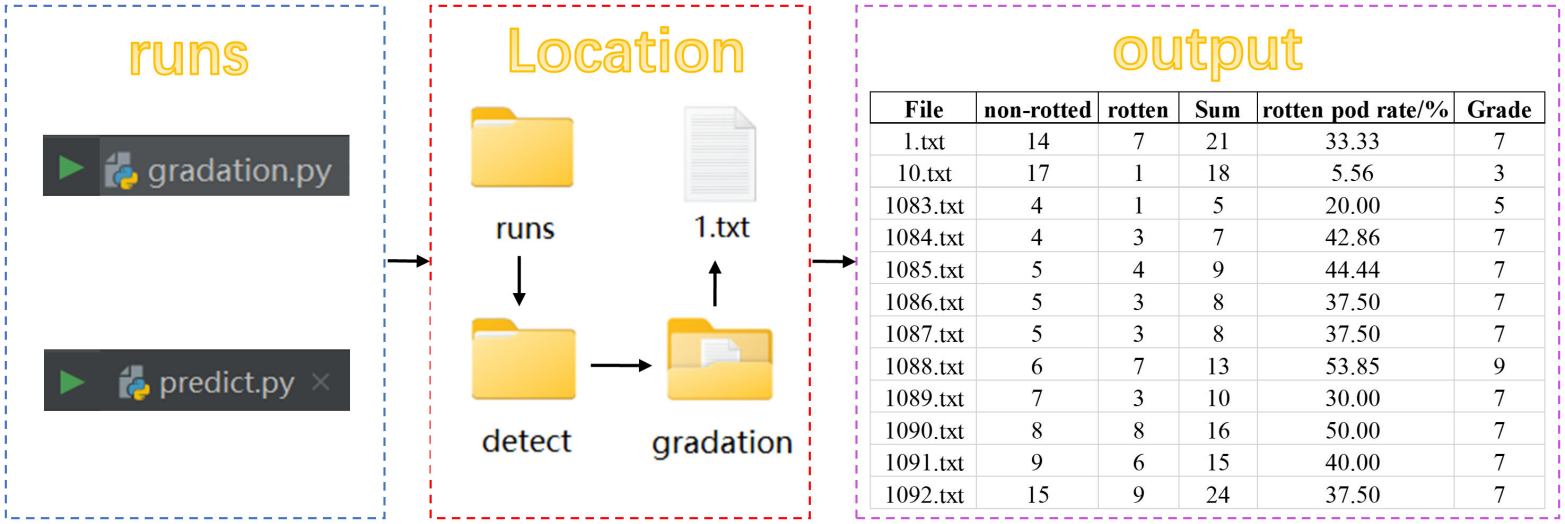
Implementing the peanut pod rot grading system in PyCharm.


As shown in **Figure 6**, an external grade classification module was put after the Prediction network to perform the grading function. After executing detect.py, the predicted images were generated in the exp folder, along with a graduation folder. This folder includes.txt files with the predicted image information, as well as statistical data on the number of non-rotted and rotten peanuts. Running gradation.py after generating the text file information will generate an.xlsx file in the root directory that contains the amount of non-rotted and rotten peanuts, as well as the overall number, rotten pod rate, and grade classification of rotten peanuts for all predicted images. The numbers of non-rotted and rotten peanuts are shown in the second and third columns, respectively. The rotten pod rate is shown in the fifth column. The grade of individual rotten peanuts, as decided by the grading criteria, is shown in the sixth column. The formula for calculating the rotten pod rate is shown in Equation (6):

(6) $$Rotten\;pod\;rate=\frac{Number\;Rotten}{Number\;Non-rotted+Number\;Rotten}$$

Where, *Number Rotten* refers to the number of rotten peanuts; *Number Non-rotted* refers to the number of non-rotted peanuts.

## 3 Results

### 3.1 Model specification

CUDA 11.3 and cuDNN8.0 were the network training environments used in this study. A 12GB NVIDIA RTX3070Ti was used as the training accelerator. Facebook’s open-source deep learning framework Python 1.11.0 was employed as the development environment, and the programming language used was Python 3.9.7. Adaptive Moment Estimation (Adam) was used to automatically modify the learning rate and solve the gradient vanishing problem, which allowed the model to converge faster and perform better. **Table 1** displays the parameter configuration of the training model.

**Table 1:** Parameter configuration of training model.

Parameter	Value
Num class	2
Epoch	200
Batch size	32
Initial learning rate	0.01

### 3.2 Evaluation indicator

This study utilized two methods, visual evaluation, and quantitative comparison, to evaluate the grading performance. Visual evaluation is a common way to visually compare and evaluate the detection results. In quantitative analysis, the evaluation indicators are Precision (P), Average Precision (AP), mean Average Precision (mAP), and Comparison Precision (CP). The calculations of the three indicators are shown in Equations (7)–(9):


(7) $$P=\frac{TP}{TP+FP}\times 100\%$$

(8) $$mAP=\frac{\sum_{n=1}^{2}AP(n)}{2}\times 100\%$$

(9) $$CP=\frac{AS}{RS}\times 100\%$$

Where, TP is the quantity of label boxes for non-rotted and rotten peanuts that accurately match the prediction boxes. FP is the number of prediction boxes containing inaccurate forecasts. P is the percentage of non-rotted and rotten peanuts that were accurately identified in each prediction box. AP represents the average Precision value of each category. mAP represents the average Precision value of all categories. AS (Automatic Statistics) represents the number of images where the model correctly identifies non-rotted and rotten peanuts in the image. RS (Realistic Statistics) represents the actual number of images of different types. CP represents the comparison precision.

### 3.3 Experiment result analysis

**Figure 7:**
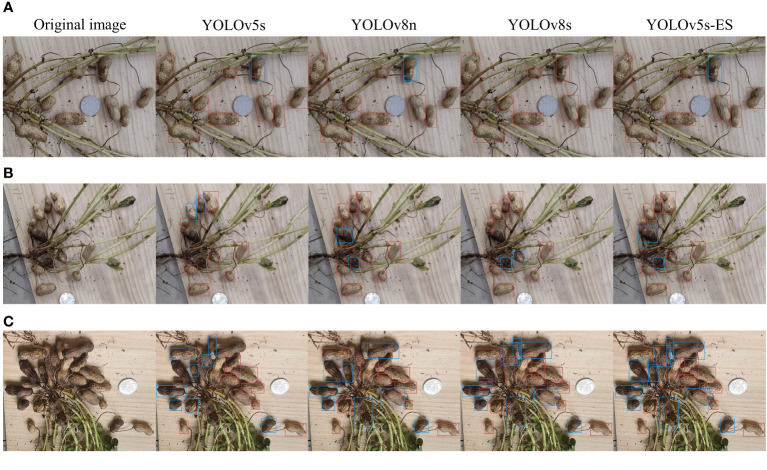
Comparison of recognition results of four models. **(A)** No adhesion; **(B)** Slight adhesion; **(C)** Severe adhesion.

Plant phenotypic detection makes extensive use of object detection. In order to compare the detection performance of YOLOv5s-ES on peanut images, this study used three YOLO-based object detection models, i.e. YOLOv5s, YOLOv8n, and YOLOv8s. Comparative experiments were carried out under the conditions of no adhesion, slight adhesion, and severe adhesion to validate the improving effect of the model. Comparative experiments aid in understanding the differences in performance between different models and drive future improvements to object detection algorithms. Simultaneously, code availability and repeatability were taken into consideration to assure the dependability and reproducibility of the experiment. **Figure 7** depicts the identification results of each model under various adhesion situations.

**Figure 7A** depicts a peanut image with no adhesion. It can be seen that the four models all had good recognition performance, achieving proper recognition with no omissions or errors. **Figure 7B** depicts a peanut image with slight adhesion, and the recognition ability of the three unimproved models all dropped. YOLOv8n missed two peanuts, and YOLOv5s missed one. Although YOLOv8s distinguished all the peanuts, the accuracy of the prediction box was low, and a single peanut pod was not marked. **Figure 7C** depicts a peanut image with severe adhesion. The identification ability of the other three models was considerably diminished, with the exception of the YOLOv5s-ES model. YOLOv8n missed 4 peanuts, with low prediction accuracy. YOLOv5s missed 3, with a relatively high accuracy of the prediction box. Although YOLOv8s recognized all the peanuts, the accuracy of the prediction box was extremely low, with cases of repeated and incorrect recognition. The YOLOv5s-ES model recognized all the peanuts correctly, with only one prediction box being inaccurately labeled. It can be concluded that the improved model YOLOv5s-ES effectively solved the problems that other three algorithms encountered when predicting images, and had the feasibility of grading peanut pod rot in practical applications.

The SA module was introduced to the YOLOv5s-ES model and the loss function CIoU was replaced with EIoU. Ablation experiments were carried out on the YOLOv5s-ES model to confirm the efficacy of the enhanced model. The experimental outcomes are displayed in **Table 2**, the mAP values represent the average results of five-fold cross-validation.

**Table 2:** Data comparison between the three enhanced models and YOLOv5s.

No.	Added SA Module	EIoU	mAP/%	P-value/%
1	×	×	86.2	/
2	√	×	88.7	0.544
3	×	√	87.5	3.759
4	√	√	92.4	0.002

The mAP of the model increased by 2.5% after the SA module was introduced to the YOLOv5s backbone network, as shown in **Table 2**. The mAP increased by 1.3% after improving the loss function of the original model. After incorporating both improvements into the model, the value of mAP reached 92.4%, 6.2% higher than that of YOLOv5s. In order to be more convincing, this study verified whether the differences between the algorithm variants were statistically significant and calculated the corresponding P-values. The results showed that the P-values of the three variants of the algorithms were less than 5%, which proved that each improvement was significantly correlated to the improvement of the detection performance. Based on this, the effectiveness of the improved model can be verified.

This study aims to enhance the performance of a peanut image recognition model, particularly under complex background conditions, through two key improvements. To assess the effectiveness of these enhancements, three groups of high-yield peanut images, which demonstrated superior recognition capabilities in preliminary experiments, were selected as cases. These images encompass rich background information and typical challenges such as mutual occlusion and environmental noise.

**Figure 8:**
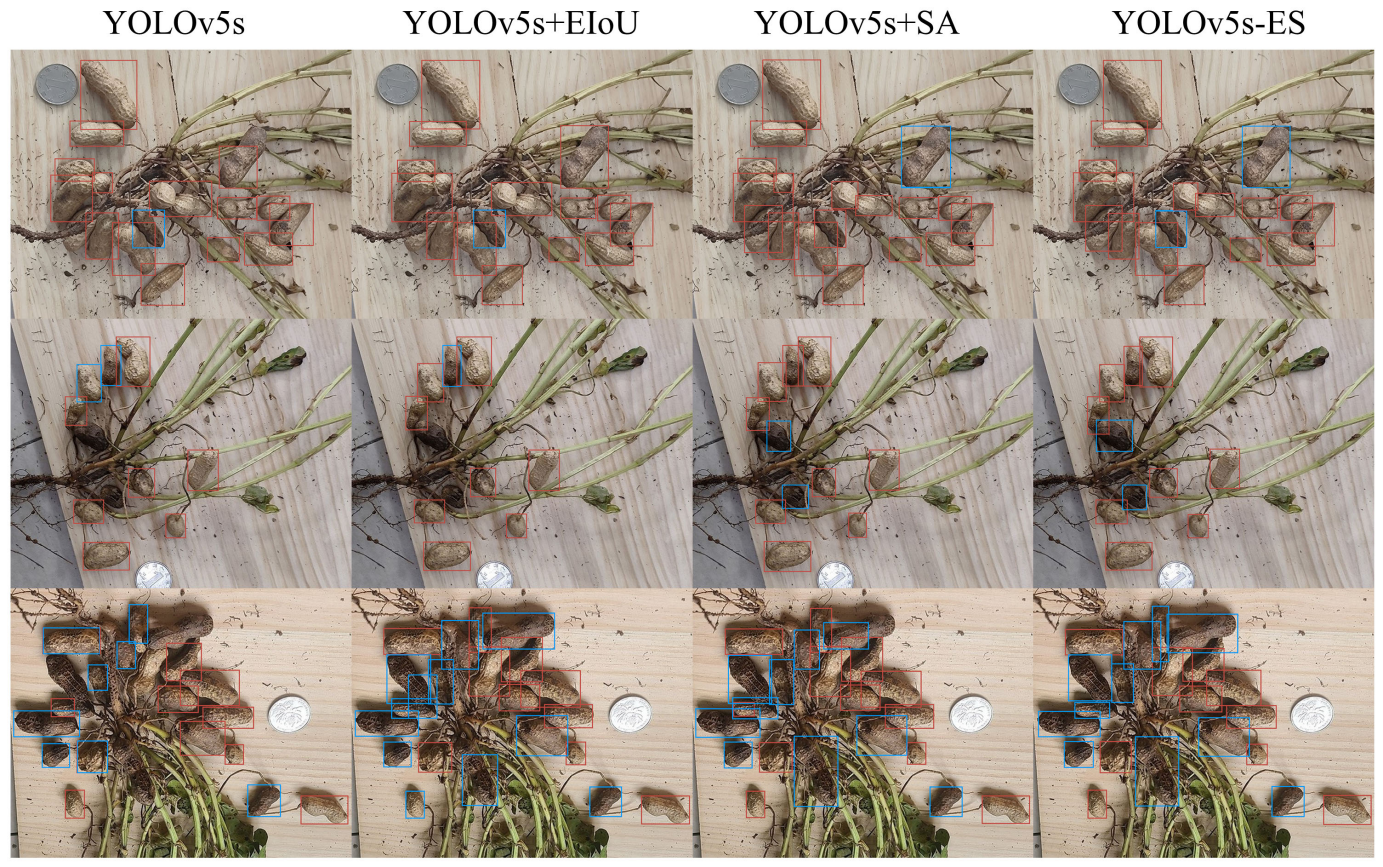
Visual comparison between the three enhanced models and YOLOv5s.

The comparison of the visualization results of the ablation experiment in **Figure 8** reveals the effectiveness of the model improvement. By integrating the SA (Spatial Attention) mechanism, the model focuses more on key areas when processing peanut images in complex backgrounds, significantly reducing the missed detection and false detection rates of the model, especially in cases where peanut leaves and roots are mixed or adhered to each other, improving the accuracy and robustness of recognition. Furthermore, the model adopts the EIoU loss function instead of the traditional IoU loss, which increases the comprehensive consideration of the target shape, size, and center point, improves the accuracy of bounding box positioning, and is crucial for the accurate classification of peanut fruit rot.

### 3.4 Comparative experiments between multiple algorithms

Based on the RS values and AS values of the four models, the CP values were calculated to validate the identification performance of the enhanced model on a solitary image. The AS value indicates the number of images in which the algorithm properly distinguished non-rotted and rotten peanuts in the image. The RS value indicates the number of images with severe adhesion, slight adhesion, and no adhesion. One hundred and fifty images of peanuts were chosen at random for the validation dataset of the experiment, with 50 images for each adhesion type. Four models - YOLOv5s, YOLOv5s-ES, YOLOv8n, and YOLOv8s - were used to identify the 150 images. The numbers of images for non-rotted and rotten peanuts that can be successfully identified via the four models were recorded as AS_1_, AS_2_, AS_3_ and AS_4_. The corresponding CP_1_, CP_2_, CP_3_ and CP_4_ were calculated as well. **Table 3** displays the comparison precision values of the four models.

**Table 3:** Comparison accuracy value comparison of different algorithms.

	YOLOv5s			YOLOv5s-ES			YOLOv8n			YOLOv8s		
	AS_1_	RS	CP_1_/%	AS_2_	RS	CP_2_/%	AS_3_	RS	CP_3_/%	AS_4_	RS	CP_4_/%
No	50	50	100	50	50	100	49	50	98	50	50	100
Slight	42	50	84	46	50	92	40	50	80	42	50	84
Severe	38	50	76	46	50	92	36	50	72	39	50	78
Total	130	150	86.67	142	150	94.67	125	150	83.33	131	150	87.33

Due to the relatively simple identification of peanut images with no adhesion, more attention was paid to comparing the prediction results of images with slight and severe adhesions. The comparison precision of the four models was 84%, 92%, 80%, and 84%, for the images with slight adhesion and 76%, 92%, 72%, and 78% for the images with severe adhesion, respectively. When it came to prediction performance, YOLOv5s-ES outperformed the three unaltered models with an improvement in the case of slight adhesion and a significant improvement in the case of severe adhesion.

**Figure 9:**
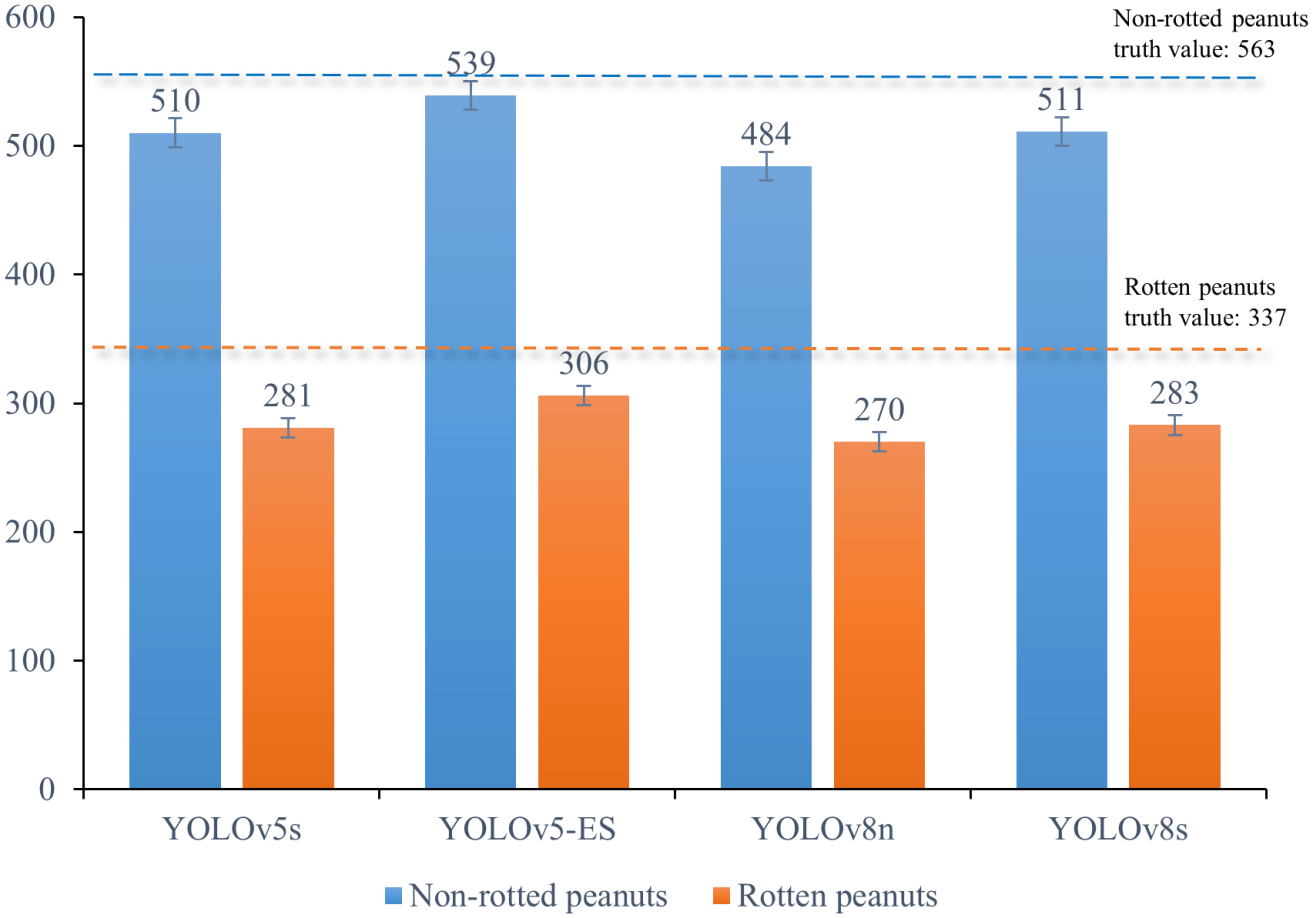
Statistical graph of non-rotted and rotten peanuts identified by four different models.

To confirm the enhanced model’s capacity to distinguish between non-rotted and rotten peanuts, 100 peanut images containing a higher proportion of rotten peanuts - a total of 563 non-rotted ones and 337 rotten ones - were chosen for identification using the four models. Statistical analysis was performed to determine how many rotten and non-rotted peanuts were identified, and the results were illustrated in **Figure 9**.

**Figure 9** illustrates that YOLOv8s identified non-rotted peanuts with a high recognition rate of 90.76%, but only 83.98% for rotten peanuts, the recognition rate of YOLOv5s is basically the same as YOLOv8s. This is due, in part, to an imbalance in the sample size between non-rotted and rotten peanuts, which limited the information available for model learning about rotten peanuts. However, some rotten pods shared coloration with rotten stems, roots, and leaves, making identification more challenging. YOLOv8n had a moderate recognition rate and a significantly weaker capacity to distinguish between rotten and non-rotted peanuts, this model had an overall recognition rate of about 83%. The above data is essentially in line with the comparison precision values listed in **Table 3**. The enhanced YOLOv5s-ES model can identify rotten peanuts with a recognition rate of 90.8% and non-rotted ones of 95.74%. The enhanced model considerably enhanced the capacity to identify rotten peanuts and had a slight improvement in identifying non-rotted ones.

**Figure 10:**
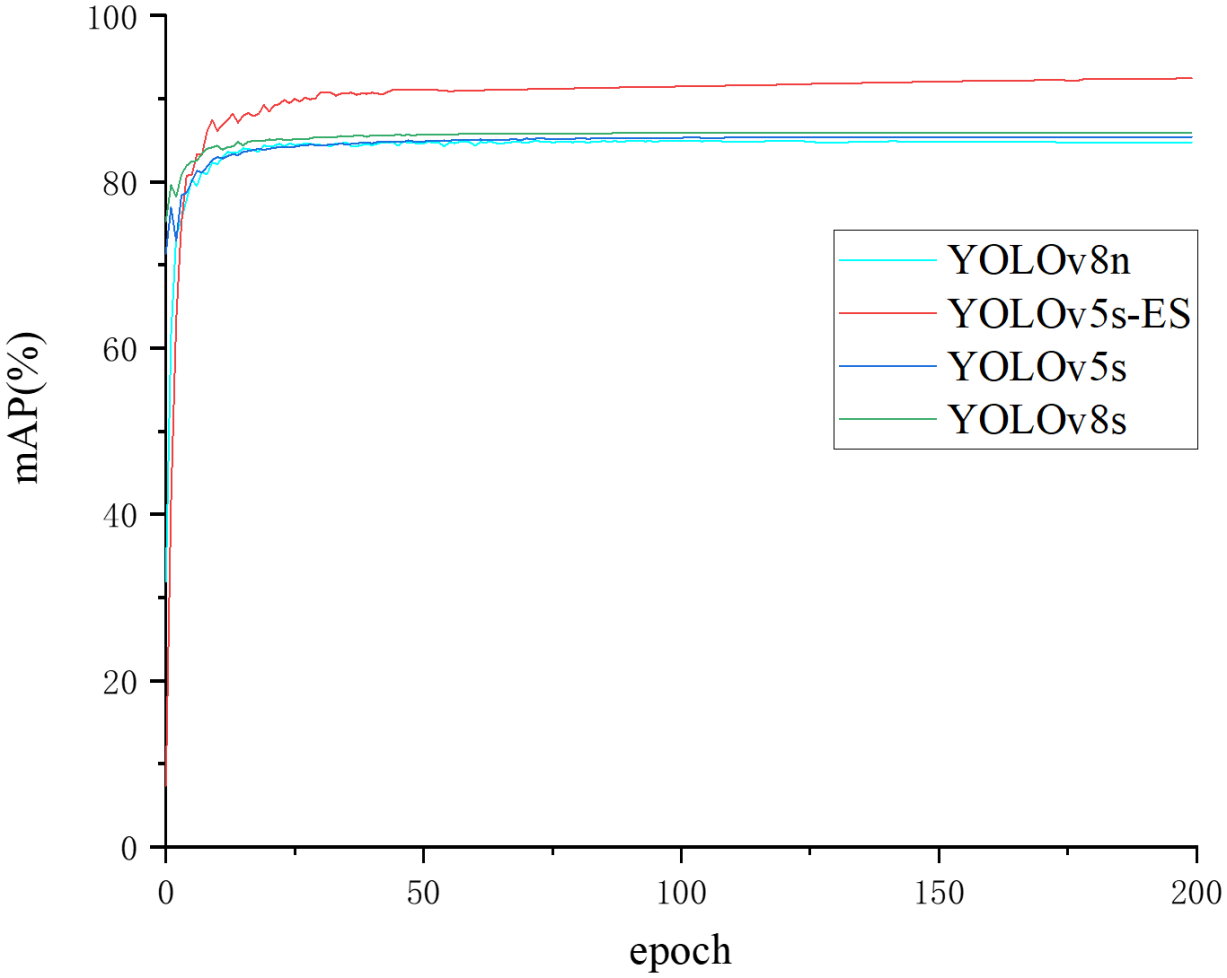
Comparison curve of mean Average Precision values of different algorithms.

To further illustrate the superiority of the algorithm proposed in this study, four models were compared for mAP change curves on the same dataset. The mAP change curve during training is displayed in **Figure 10**. It can be seen that YOLOv5s, YOLOv8n, YOLOv8s, and YOLOv5s-ES had mAP values of 85.7%, 84.7%, 85.9%, and 92.4%, respectively. The convergence rates of all four curves were incredibly quick, and the three unimproved models achieved fitting with around 75 epochs. Excessive data fitting may result in unstable model parameters. When there is some randomness or fluctuation in the data, the model may update parameters excessively to accommodate these changes, resulting in inconsistent model performance. Instability may affect the model’s reliability and interpretability, resulting in poor performance in practical applications since it cannot catch potential patterns and overall trends in the data. After 100 epochs, the mAP of YOLOv5s-ES hit 91.4% and tended to stabilize, eventually achieving 92.4%. It can be concluded that the enhanced model leveraged the likelihood of capturing real patterns and overall trends in the data, rather than unnecessarily responding to the noise and intricacies of the training data. In this way, the generalization ability of the model can be promoted on unknown data, making it more suitable for practical applications.

**Figure 11:**
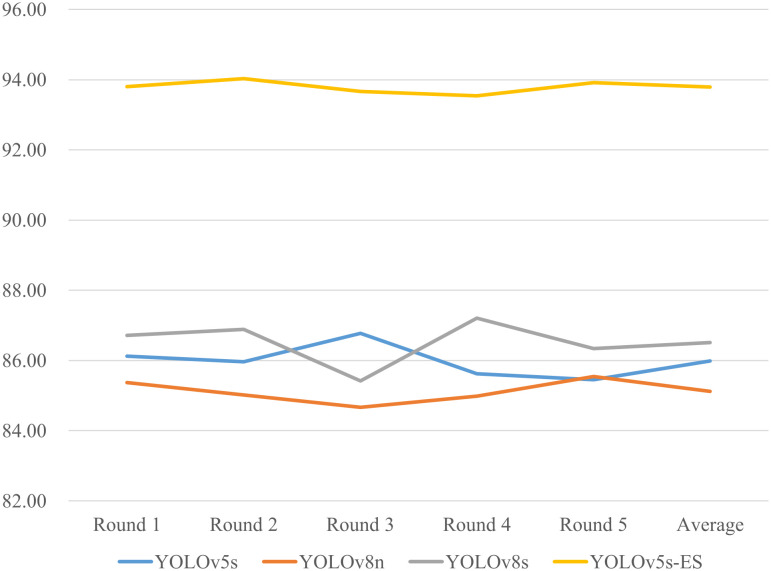
Comparison of Precision (%) for five-fold cross-validation of four models.

**Figure 12:**
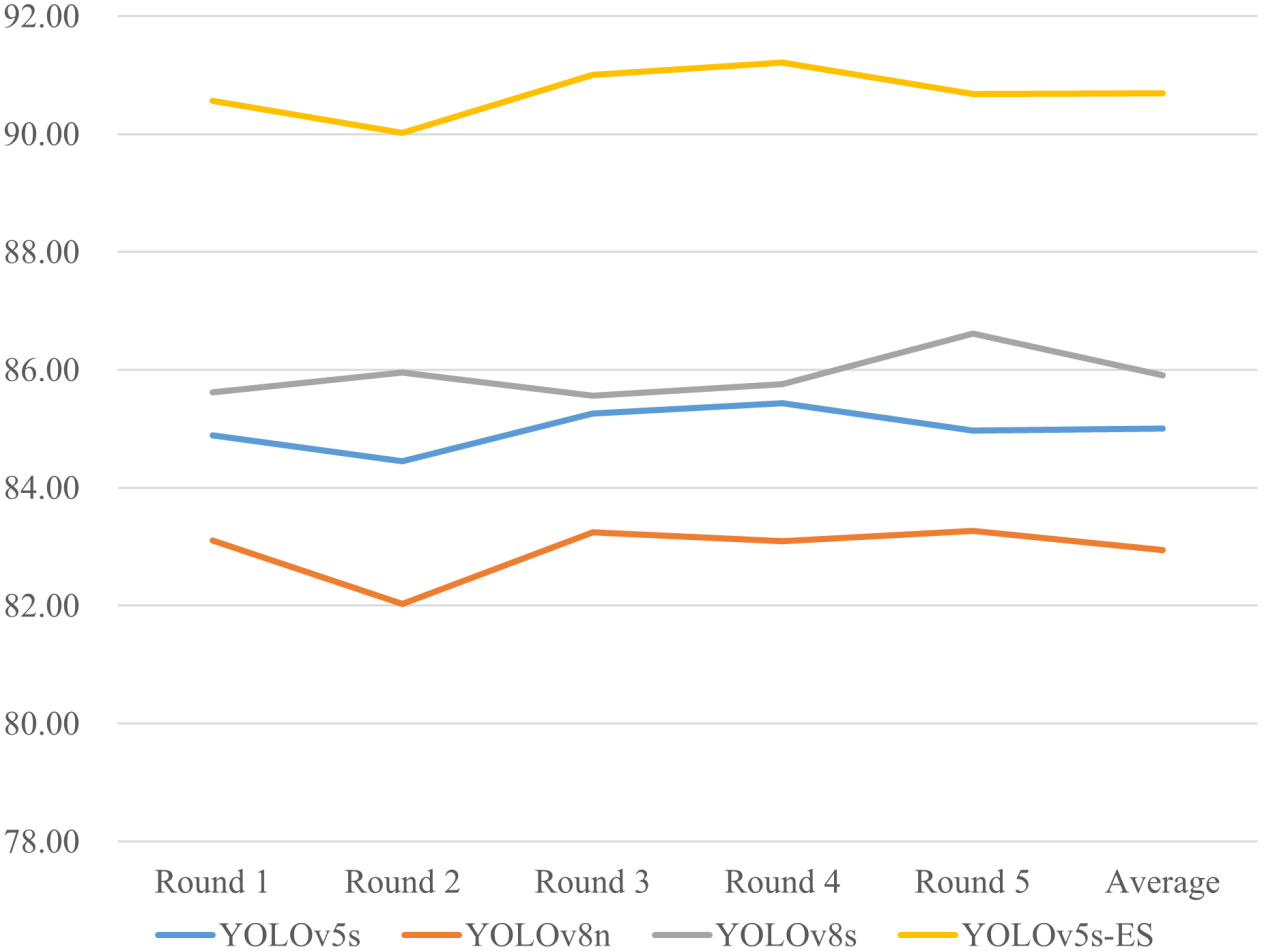
Comparison of Recall (%) for five-fold cross-validation of four models.

To address the potential inaccuracies in assessment results that might arise from a single dataset split, a five-fold cross-validation study was conducted on four different models. Precision and Recall values from five separate trials were collected and averaged. The results of the five-fold cross-validation for both metrics are presented in **Figures 11** and **12**. The data in the figures reveal only minor fluctuations in the model’s recognition capabilities across the five randomly partitioned datasets, confirming the model’s robust generalization performance in identifying peanut fruit rot disease. The Precision of the improved model YOLOv5s-ES was 93.8%, 7.8%, 8.7%, and 7.3% higher than YOLOv5s, YOLOv8n, and YOLOv8s, respectively. The Recall value was 90.7%, which increased by 5.7%, 7.7%, and 4.8% than the other three models, respectively. As shown in **Table 4**.

**Table 4:** Comparison of precision and recall metrics across four models using five-fold cross-validation.

Model	YOLOv5s	YOLOv5s-ES	YOLOv8n	YOLOv8s
Precision (%)	86.0	93.8	85.1	86.5
Recall (%)	85.0	90.7	83.0	85.9

## 4 Discussions

Peanut pod rot causes fruit degradation and yield loss, making prevention and management difficult and potentially transmittable. Grade classification of peanut pod rot allows for the evaluation of disease resistance, the selection of outstanding germplasm resources, and the promotion of breeding improvement. This study suggests an object detection approach based on YOLOv5s-ES in response to the drawbacks of manual classification, which can successfully increase the efficacy and precision of pod rot grading and eventually replace conventional manual classification. Although this study is of great significance in addressing pod rot grading, there are certain concerns that require additional research and analysis.

**Figure 13:**
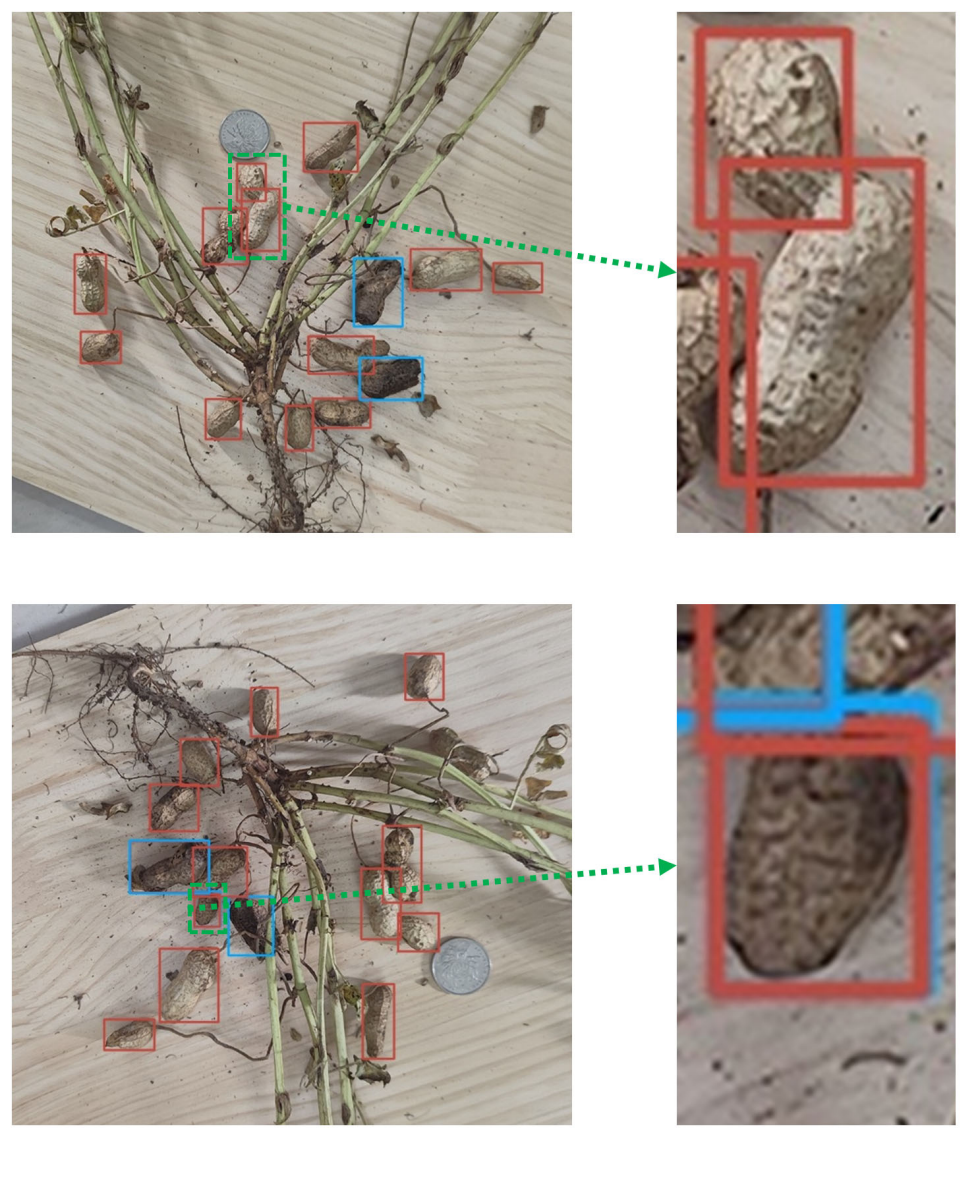
Two types of misidentification present in the improved model.

The improved YOLOv5s-ES model may encounter misidentification during prediction. Two typical examples are shown in **Figure 13**.

In **Figure 13A**, the model accurately identified and labeled the rotten peanut, but incorrectly identified the peanut as a non-rotted one and repeated labeling, leaving the model unable to differentiate between the non-rotted and rotten types of the peanut. One reasonable explanation on the one hand is the insufficient debugging of the model parameter threshold, which makes it hard for the model to reliably identify whether this type of peanut belongs to non-rotted or rotten. Based on this, improvement can be achieved through parameter adjustment, threshold modification, etc. On the other hand, some peanut pods have a moderate degree of decay, making it hard to distinguish between the non-rotted and rotten types solely based on phenotypic sampling. In this case, semantic segmentation methods can be introduced. Specifically, the diseased area of each peanut is calculated, the proportion of which can be used to determine whether the pod belongs to a rotten one. In this way, the problem can be solved using the judgment results of semantic segmentation combined with object detection algorithms.

In **Figure 13B**, a peanut pod was mistakenly identified as two pods, meaning that the model labeled a valencia type peanut as a double-kernal one and a single-kernal one during prediction. This error tends to happen when the sample size is insufficient. During training, the model identified a small number of valencia type peanuts, so that when new valencia type peanuts appeared, the entire pod could not be correctly identified and was misjudged as two or more double-kernal and single-kernal pods. Increasing the sample size, especially the images of valencia type peanuts, is an effective way to solve such recognition errors.

Furthermore, after being infected with peanut pod rot, some peanut pods only form a thin coating of decay on the surface, leaving the kernels unaffected. As a result, the impact on peanut yield includes the rotten kernel rate. The degree of pod rot was used to classify peanut pod rot in this study, and the rotten kernel rate was not considered. As a result, the projected data has a poor practical application value in yield estimation, which is a shortcoming of machine vision-based pod rot grade classification. In order to ensure that the design scheme can be used effectively in more aspects, greater attention may be paid to the grading of peanut pod rot under the dual factors of rotten pod rate and rotten kernel rate.

Moreover, due to the differences in pod rot among various peanut varieties and the lack of relevant samples, this study cannot predict whether the model’s recognition capability for images of other peanut varieties will decrease. In order to overcome the aforementioned drawbacks, we will expand the sample size of different kinds of peanuts, conduct transfer learning across different varieties with the model, combine semantic segmentation methods, and enhance the model’s performance. First, we will ascertain whether a single peanut has pod rot. Then, the peanut pods in the image will be annotated using object identification methods to improve the accuracy of the results. To further increase prediction accuracy and visibility, it is feasible to introduce an instance segmentation algorithm and confirm its benefits in extreme peanut adhesion scenarios. Additionally, data on peanut pod rot in complex environments should be analyzed concurrently to strengthen the resilience of the model and make it more applicable to peanut plants in various conditions and cultivars.

## 5 Conclusions

Starting with the relevance of grading individual peanut pod rot, this study employed the Jinongxian No.1 peanut as the experimental object in the field management planting base. To address the inadequacies of the current grade classification for peanut pod rot, a machine vision-based method was proposed using a modified loss function and feature extraction backbone network of the YOLOv5s algorithm.

(1) The SA module was introduced to the YOLOv5s network as the main framework to overcome problems like adhesion and obstruction in the dense development of certain peanut plants, which are vulnerable to interference from roots, stems, and leaves. The feature extraction ability of the network for identifying non-rotted and rotten peanuts was enhanced by substituting the EIoU for the CIoU in the original network in response to the sample imbalance problem caused by the fact that the number of non-rotted pods is much higher than the number of rotten pods in actual situations.

(2) With a Precision value of 93.8%, the improved model YOLOv5s-ES outperformed YOLOv5s, YOLOv8n, and YOLOv8s by 7.8%, 8.4%, and 7.3%, respectively. Its mAP value was 92.4%, outperforming YOLOv5s, YOLOv8n, and YOLOv8s by 6.7%, 7.7%, and 6.5%, respectively. With a non-rotted pods recognition rate of 95.74% and a rotten pods recognition rate of 90.8%, the comparison precision reached 94.67%, satisfying the requirements of exact recognition.

(3) With the addition of a grade classification module after the Prediction network, this study realized the calculation of the number of non-rotted and rotten peanuts as well as the rotten pod rate in the images. The results were then written into a.txt file. The grading of pod rot can be completed by adding the grade classification module to the YOLOv5s-ES model, which allows the database to read text files and record the number of non-rotted and rotten peanuts, the rotten pod rate, and the grading of pod rot.

In conclusion, the improved model proposed in this study will help the automatic grade classification of individual peanut pod rot in practical prediction applications, facilitating in the screening of superior germplasm resources and peanut breeding.

## Data availability statement

The dataset supporting this study can directly download using the link below: https://github.com/JiaLBYGG/Peanut-pod-rot-dataset.git.

## Author contributions

YL: Writing – original draft, Validation, Methodology, Conceptualization. XL: Writing – review & editing. YF: Writing – original draft, Validation, Project administration, Data curation, Conceptualization. LL: Writing – review & editing, Supervision, Funding acquisition. LS: Writing – review & editing, Visualization, Supervision, Funding acquisition. GY: Writing – original draft, Investigation, Formal analysis, Data curation. YG: Writing – original draft, Investigation, Data curation. YZ: Writing – original draft.
